# Impact of the interval between neoadjuvant immunotherapy and surgery on prognosis in esophageal squamous cell carcinoma (ESCC): a real-world study

**DOI:** 10.1007/s00262-024-03787-2

**Published:** 2024-08-06

**Authors:** Guozhen Yang, Yutong Hong, Xiaomin Zhang, Chufeng Zeng, Linyu Tan, Xu Zhang

**Affiliations:** 1 Department of Thoracic Oncology, Sun Yat-Sen University Cancer Center, Guangzhou, China; 2 Guangdong Esophageal Cancer Institute, Guangzhou, China; 3 State Key Laboratory of Oncology in South China, Guangdong Provincial Clinical Research Center for Cancer, Sun Yat-Sen University Cancer Center, Guangzhou, 510060 People’s Republic of China; 4 School of Nursing, Sun Yat-Sen University, Guangzhou, China

**Keywords:** Esophageal squamous cell carcinoma, Immunotherapy, Interval, Pathological complete response, Prognosis

## Abstract

**Background:**

The time interval between neoadjuvant immunotherapy and surgery is 6 weeks for esophageal squamous cell carcinoma (ESCC), but whether delayed surgery affects prognosis remains unclear.

**Methods:**

Clinical data of locally advanced ESCC who underwent neoadjuvant immunotherapy followed by esophagectomy from November 2019 to December 2022 were collected. The surgery outcomes and prognosis were compared between short-interval (time to surgery ≤ 6 weeks) and long-interval groups (time to surgery > 6 weeks).

**Results:**

152 patients were enrolled totally, with a ratio of 91:61 between short-interval and long-interval groups. The rate of pathological complete response in the short-interval and long-interval groups were 34.1% and 24.6% (*P* = 0.257). Delayed surgery did not have a significantly impact on the number of lymph node dissections (*P* = 0.133), operative duration (*P* = 0.689), blood loss (*P* = 0.837), hospitalization duration (*P* = 0.293), chest drainage duration (*P* = 0.886) and postoperative complications (*P* > 0.050). The 3-year Overall survival (OS) rates were 85.10% in the short-interval group, and 82.07% in the long-interval group (*P* = 0.435). The 3-year disease-free survival (DFS) rates were 83.41% and 70.86% in the two groups (*P* = 0.037). Subgroup analysis revealed that patients with a favorable response to immunotherapy (tumor regression grade 0) exhibited inferior 3-year OS (long-interval vs. short-interval: 51.85% vs. 91.08%, *P* = 0.035) and DFS (long-interval vs. short-interval: 47.40% vs. 91.08%, *P* = 0.014) in the long-interval group.

**Conclusions:**

Delayed surgery after neoadjuvant immunotherapy does not further improve pathological response; instead, it resulted in a poorer DFS. Especially for patients with a favorable response to immunotherapy, delayed surgery increases the risk of mortality and recurrence.

**Supplementary Information:**

The online version contains supplementary material available at 10.1007/s00262-024-03787-2.

## Introduction

Esophageal cancer ranks as the seventh most common malignant neoplasm worldwide and the sixth principal cause of cancer-induced mortality. According to the 2020 GLOBOCAN statistics [1], there are approximately 604,000 new cases of esophageal cancer worldwide each year, with around 544,000 new deaths. The incidence and mortality rates remain consistently elevated. China is identified as a high-incidence country for esophageal cancer, with esophageal squamous cell carcinoma (ESCC) constituting over 90% of cases. [2, 3]. The bulk of cases are typically identified at an locally advanced stage, due to the propensity for early lymph node metastasis.

In recent years, programmed death protein 1 (PD-1) antibodies plus neoadjuvant chemotherapy has been investigated in several clinical trials for locally advanced ESCC [4, 5]. This neoadjuvant regimen has demonstrated the potential to reduce the size of primary tumors, increase the success rate of surgical resection, achieve favorable rates of pathological complete response (pCR), and exhibit tolerable and manageable toxicity profiles. It offers promising prospects for widespread application in locally advanced ESCC. However, patient's condition after neoadjuvant treatment, local lesion and the systemic inflammatory response, can all impact surgical results. These factors may change over time, indicating that appropriate surgical timing is crucial for optimizing surgical outcomes. Current literature on the timing of surgery following neoadjuvant therapy shows inconsistent conclusions. Kim et al. [6] and Tessier et al. [7] reported that delayed surgery did not affect prognosis, whereas Qin et al. [8] observed that delayed surgery was associated with worse prognosis. Currently, most clinical trials performed surgery within 6 weeks after the end of neoadjuvant immunochemotherapy [9–11], which was based on the experience from traditional neoadjuvant chemoradiotherapy [12]. In traditional neoadjuvant chemoradiotherapy, surgery is typically scheduled within 4–6 weeks after radiotherapy to avoid exacerbating tissue fibrosis induced by radiation and increasing the surgical difficulty [13]. However, PD-1 inhibitor exert completely different mechanisms of action from radiation therapy, by obstructing the interaction between PD-1 and programmed death-ligand 1 (PD-L1), thus alleviating the suppression of T cells, and restoring their capacity to combat tumors. [14–16]. Additionally, the durable response of immunotherapy could promote tumor continue to shrink even after the drug is stopped for a period of time [17]. Therefore, it remains unclear whether delaying surgery in the context of immunotherapy would impact patient prognosis.

Therefore, we conducted a population-based, real-world, retrospective study, which enrolled patients with locally advanced ESCC who underwent neoadjuvant PD-1 inhibitor plus chemotherapy followed by surgery. The patient cohort was divided into short-interval group (time to surgery ≤ 6 weeks) and long-interval group (time to surgery > 6 weeks), to assess whether delayed surgery after immunotherapy affected surgical and pathological outcomes as well as long-term prognosis.

## Methods

### Patients selection

We retrospectively collected patients with ESCC who underwent neoadjuvant PD-1 inhibitor combined chemotherapy followed by surgery at the Department of Thoracic Surgery, Sun Yat-sen University Cancer Center from November 2019 to December 2022. Inclusion criteria were as follows: 1) age ranging from 18 to 80 years; 2) pathologically diagnosed with thoracic ESCC; 3) Clinical stage T1N1-3 or T2-4aN0-3 according to the Union for International Cancer Control/American Joint Committee on Cancer (UICC/AJCC), 8th edition; 4) administration of at least one cycle of PD-1 inhibitor combined chemotherapy prior to surgery; 5) availability of complete clinical, pathological, and follow-up data; 6) eastern cooperative oncology group (ECOG) performance status of 0–1. Exclusion criteria were: (1) adenocarcinoma, sarcoma, or other non-squamous cell carcinoma types; (2) received other neoadjuvant regimen before surgery, such as radiotherapy, targeted therapy; (3) clinical data missing.

### Neoadjuvant regimen

In the overall cohort, the median number of neoadjuvant treatment cycles was 3 (range: 2–6). PD-1 inhibitors used in the study comprised camrelizumab, pembrolizumab, sintilimab, tislelizumab, toripalimab, and penpulimab. Chemotherapy regimens consisted of: (1) paclitaxel-based drugs plus platinum-based regimens, and (2) paclitaxel-based drugs plus fluoropyrimidine-based regimens. Detailed treatment protocols were provided in Supplementary Figure 1. Drug dosage and administration details were outlined in Supplementary Table 1.

### Surgery procedure

Surgery was conducted after completing neoadjuvant therapy. The interval between neoadjuvant treatment and surgery was defined as the duration from the last cycle of treatment to the surgical procedure. Surgical approaches included traditional thoracotomy, McKeown or Ivor-Lewis minimally invasive esophagectomy (MIE), and robotic-assisted thoracoscopic/laparoscopic esophagectomy. Standard two-field lymph node dissection was conducted, with consideration of three-field lymph node dissection if preoperative cervical lymph node ultrasound indicated cervical lymph node metastasis. All surgical procedures were conducted by experienced thoracic surgeon.

### Follow-up

The initial postoperative follow-up occurred at 3 months post-surgery, followed by subsequent visits every 3 months during the first two years, and thereafter at intervals of 6–12 months. Follow-up included contrast-enhanced computed tomography (CT) scans of the chest and abdomen, along with ultrasonography of the supraclavicular lymph nodes. If dysphagia or anastomotic leakage was suspected, electronic gastroscopy or endoscopic ultrasound (EUS) was performed. Positron emission tomography/computed tomography (PET-CT) was conducted if distant metastasis was suspected.

### Study endpoint

The primary endpoints of the study comprised disease-free survival (DFS) and overall survival (OS), with secondary endpoints encompassing pathological complete response (pCR), objective response rate (ORR), primary tumor regression, lymph node regression, perioperative complications, and patterns of recurrence. DFS was delineated as the duration from surgery to the occurrence of disease recurrence, metastasis, or mortality, while OS was delineated as the duration from surgery to death from any cause. Evaluation of efficacy adhered to the Response Evaluation Criteria in Solid Tumors (RECIST) criteria (version 1.1). Complete response (CR) denoted the disappearance of all target lesions. Partial response (PR) denoted a reduction in the sum of the longest diameters of all target lesions by 30% or more. Progressive disease (PD) denoted an increase in the sum of the longest diameters of all target lesions by at least 20%. Stable disease (SD) denoted a response between PR and PD. ORR was defined as the proportion of patients achieving CR or PR. Preoperative clinical TNM staging was conducted for all patients via imaging examination. Postoperative pathological TNM staging determined according to pathological findings. Staging adhered to the 8th edition of the UICC/AJCC TNM staging system.

### Statistical analysis

Categorical variables were depicted as frequencies (%). Continuous variables following a normal distribution were expressed as mean ± standard deviation, whereas those not following a normal distribution are presented as median (IQR). The Chi-square test or Fisher’s exact test was employed for comparisons among categorical variables. The t-test or Wilcoxon test was utilized for comparisons among continuous variables. To mitigate the impact of clinical factors on endpoints, multivariable logistic regression models or multiple linear regression models were employed to adjust for age, sex, tumor location, differentiation, smoking history, alcohol consumption history, ECOG performance status, clinical T stage, clinical N stage, clinical TNM stage, cycles of neoadjuvant treatment, and the regimen of chemotherapy. The Kaplan–Meier method was utilized to estimate DFS and OS, with survival differences compared using the Log-rank test. Univariate and multivariate Cox regression models were applied to analyze the influence of clinical factors on prognosis. *P* value < 0.05 (both sides) was deemed statistically significant. All analyses were conducted using R version 4.0.4 (R Foundation for Statistical Computing, Vienna, Austria).

## Results

### Baseline characteristics

From November 2019 to December 2022, 152 patients diagnosed with locally advanced ESCC underwent radical esophagectomy following neoadjuvant PD-1 inhibitor combined with chemotherapy at the Department of Thoracic Surgery of the Sun Yat-sen University Cancer Center. The median age of the included patients was 58 years (range: 41.0–78.0 years). All patients underwent clinical staging by chest enhanced CT scans and endoscopic examinations, with the majority being stage II-III (stage II: 36 cases, 23.7%; stage III: 90 cases, 59.2%). The median interval from the completion of the last neoadjuvant treatment to surgery was 5.71 weeks (range: 1.29–25.0 weeks). There were 91 cases in short-interval group (time to surgery ≤ 6 weeks), with a median time to surgery of 4.71 weeks (range: 1.29–6.0 weeks), and 61 cases in long-interval group (> 6 weeks), with a median time to surgery of 7.29 weeks (range: 6.14–25.0 weeks). The clinical baseline characteristics of the two groups were depicted in Table 1. Short-interval group and long-interval group were well-balanced and comparable in terms of sex (*P* = 0.264), age (*P* = 0.100), tumor location (*P* = 0.323), smoking history (*P* = 0.256), alcohol consumption history (*P* = 0.286), body mass index (BMI) (*P* = 0.927), ECOG performance status (*P* = 0.195), histological differentiation (*P* = 0.942), clinical T stage (*P* = 0.470), clinical N stage (*P* = 0.486), clinical TNM stage (*P* = 0.315), surgical approach (*P* = 0.615), and cycles of neoadjuvant treatment (*P* = 0.830).

**Table 1:** Baseline characteristics of ESSS

Characteristics	Total (n = 152)	Short-interval group (n = 91)	Long-interval group (n = 61)	*P* value
Sex				0.264
Male	128 (84.2%)	74 (81.3%)	54 (88.5%)	
Female	24 (15.8%)	17 (18.7%)	7 (11.5%)	
Age (years old)				0.100
Mean	59.5	59.5	61.7	
Median (Min, Max)	58.0 (41.0, 78.0)	58.0 (41.0, 78.0)	63 (31, 80)	
Tumor location				0.323
Upper thoracic	9 (5.9%)	4 (4.4%)	5 (8.2%)	
Middle thoracic	94 (61.9%)	54 (59.3%)	40 (65.6%)	
Lower thoracic	49 (32.2%)	33 (36.3%)	16 (26.2%)	
Smoking history				0.256
Yes	99 (65.1%)	56 (61.5%)	43 (70.5%)	
No	53 (34.9%)	35 (38.5%)	18 (29.5%)	
Alcohol consumption history				0.286
Yes	57 (37.5%)	31 (34.1%)	26 (42.6%)	
No	95 (62.5%)	60 (65.9%)	35 (85.4%)	
BMI (kg/m2)				0.927
≦18.5	24 (15.8%)	14 (15.4%)	10 (16.4%)	
> 18.5 and ≦23.9	95 (62.5%)	58 (63.7%)	37 (60.7%)	
> 23.9	33 (21.7%)	19 (20.9%)	14 (22.9%)	
ECOG				0.195
0	99 (65.1%)	63 (69.2%)	36 (59.0%)	
1	53 (34.9%)	28 (30.8%)	25 (41.0%)	
Differentiation				0.942
Well differentiated	7 (4.6%)	4 (4.4%)	3 (4.9%)	
Moderately differentiated	108 (71.1%)	64 (70.3%)	44 (72.1%))	
Poorly differentiated	37 (24.3%)	23 (25.3%)	14 (23.0%)	
Clinical stage T				0.470
T1	1 (0.6%)	1 (1.1%)	0 (0.0%)	
T2	41 (27.0%)	26 (28.6%)	15 (24.6%)	
T3	100 (65.8%)	60 (65.9%)	40 (65.6%)	
T4	10 (6.6%)	4 (4.4%)	6 (9.8%)	
Clinical stage N				0.486
N0	17 (11.2%)	13 (14.3%)	4 (6.6%)	
N1	63 (41.4%)	37 (40.6%)	26 (42.6%)	
N2	53 (34.9%)	31 (34.1%)	22 (36.1%)	
N3	19 (12.5%)	10 (11.0%)	9 (14.7%)	
Clinical stage TNM				0.315
I	1 (0.7%)	1 (1.1%)	0 (0.0%)	
II	36 (23.7%)	25 (27.5%)	11 (18.0%)	
III	90 (59.2%)	53 (58.2%)	37 (60.7%)	
IV	25 (16.4%)	12 (13.2%)	13 (21.3%)	
Surgical approach				0.615
MIE	146 (96.1%)	88 (96.7%)	58 (95.1%)	
Thoracotomy	6 (3.9%)	3 (3.3%)	3 (4.9%)	
Cycles of neoadjuvant treatment				0.830
≤ 2	36 (23.7%)	21 (23.1%)	15 (24.6%)	
> 2	116 (76.3%)	70 (76.9%)	46 (75.4%)	
Adjuvant treatment				
Yes	9 (5.9%)	5 (5.5%)	4 (6.6%)	> 0.999
No	143 (94.1%)	86 (94.5%)	57 (93.4%)	

### Clinical effect and pathological outcomes

The clinical effect and pathological outcomes were shown in Table 2. After neoadjuvant PD-1 inhibitor combined with chemotherapy, 8.8% and 3.3% of patients in the short-interval and long-interval groups achieved CR (OR: 0.424, 95%CI: 0.097–1.851,* P* = 0.254), 65.9% and 73.8% of patients achieved PR (OR: 1.926, 95%CI: 0.844–4.396, *P* = 0.120), with no statistically significant differences between the two groups.

**Table 2:** The clinical efficacy between short-interval group and long-interval group

	Short-interval group (n = 91)	Long-interval group (n = 61)	Before adjust		After adjust*	
			OR (95%CI)	*P* value	OR (95%CI)	*P* value
CR	8 (8.8%)	2 (3.3%)	0.352 (0.073–1.531)	0.317	0.424 (0.097–1.851)	0.254
PR	60 (65.9%)	45 (73.8%)	1.453 (0.717–2.975)	0.372	1.926 (0.844–4.396)	0.120
SD	21 (23.1%)	13 (21.3%)	0.903 (0.415–1.955)	0.845	0.608 (0.252–1.464)	0.267
PD	2 (2.2%)	1 (1.6%)	0.742 (0.050–6.500)	> 0.999	0.118 (0.003–4.910)	0.261
ORR	68 (74.7%)	47 (77.1%)	1.136 (0.547–2.346)	0.848	1.020 (0.442–2.356)	0.963
T downstaging	63 (69.2%)	42 (68.9%)	0.983 (0.491–1.946)	1.000	0.993 (0.438–2.250)	0.986
N downstaging	64 (70.3%)	45 (73.8%)	1.187 (0.570–2.468)	0.715	1.025 (0.404–2.603)	0.958
TNM downstaging	65 (71.4%)	43 (70.5%)	0.956 (0.466–1.906)	1.000	0.980 (0.444–2.166)	0.961
pCR	31 (34.1%)	15 (24.6%)	0.631 (0.299–1.297)	0.280	0.618 (0.269–1.421)	0.257

*The multivariate logistic regression model adjusted age, sex, tumor location, differentiation, smoking history, alcohol consumption history, ECOG performance status, clinical T stage, clinical N stage, clinical TNM stage, cycles of neoadjuvant treatment and the regimen of chemotherapy

In terms of pathological outcomes, the proportions achieving pCR in the short-interval group and long-interval group were 34.1% and 24.6%, respectively. After adjustment, still no statistically significant disparity was observed between the two groups (OR: 0.618, 95%CI: 0.269–1.421, *P* = 0.257). Postoperative pathological results were compared with preoperative clinical staging. 69.2% and 68.9% of patients in the short-interval group and long-interval group experienced T downstaging (OR: 0.983, 95%CI: 0.491–1.946, *P* = 1.000), while 70.3% and 73.8% of patients experienced N downstaging (OR: 1.187, 95%CI: 0.570–2.468, *P* = 0.715). Regarding overall TNM staging, 71.4% and 70.5% of patients in the short-interval group and long-interval group, respectively, showed downstaging after neoadjuvant treatment, with no statistically significant difference between the two groups (OR: 0.956, 95%CI: 0.466–1.906, *P* = 1.000). Considering the impact of clinical factors and control for potential biases, adjustments were made for sex, age, tumor location, smoking status, drinking status, ECOG performance score, differentiation, clinical T stage, clinical N stage, clinical TNM stage, cycles of neoadjuvant treatment and regimen of chemotherapy. The results after adjustments still revealed that the interval between neoadjuvant immunotherapy and surgery had no significant impact on primary tumor and lymph node downstaging.

### Adverse events of neoadjuvant treatment

The adverse effects of neoadjuvant treatment between short-interval and long-interval groups were displayed in Table 3. In short-interval group, 50.5% of patients experienced at least one adverse events, with the most common adverse events being anemia (39.6%), thyroid dysfunction (13.2%), increased alanine transaminase (11.0%), leukopenia (8.8%) and neutropenia (8.8%). In long-interval group, 59.0% of patients experienced at least one adverse events, with the most common being anemia (41.0%), leukopenia (16.4%), increased alanine transaminase (16.4%), neutropenia (13.1%) and thyroid dysfunction (9.8%). There was no statistically significant difference in the incidence of adverse events between the two groups.

**Table 3:** The adverse effects between short-interval group and long-interval group

	Short-interval group (n = 91)	Long-interval group (n = 61)	Before adjust		After adjust*	
			OR (95%CI)	*P* value	OR (95%CI)	*P* value
Total	46 (50.5%)	36 (59.0%)	1.409 (0.727–2.641)	0.320	1.158 (0.561–2.388)	0.692
Leukopenia	8 (8.8%)	10 (16.4%)	2.034 (0.778–5.524)	0.201	1.578 (0.430–5.793)	0.492
Neutropenia	8 (8.8%)	8 (13.1%)	1.566 (0.540–4.527)	0.428	1.167 (0.252–1.464)	0.819
Anemia	36 (39.6%)	25 (41.0%)	1.061 (0.562–2.094)	0.868	1.228 (0.575–2.623)	0.596
Increased alanine transaminase	10 (11.0%)	10 (16.4%)	1.588 (0.591–4.257)	0.340	2.339 (0.711–7.692)	0.162
Thyroid dysfunction	12 (13.2%)	6 (9.8%)	0.718 (0.254–2.032)	0.615	0.848 (0.259–2.779)	0.785

### Surgery outcomes

As presented in Table 1 in Supplementary material 2, both groups of patients achieved R0 resection. There were no statistically significant differences between short-interval group and long-interval group in terms of the number of lymph nodes dissected (40.34 ± 16.77 vs. 44.51 ± 21.13, *P* = 0.133), positive lymph node counts (0.70 ± 1.82 vs. 0.74 ± 1.28, *P* = 0.887), operative time (325.13 ± 141.48 min vs. 340.30 ± 191.10 min, *P* = 0.689), blood loss (100 ml vs. 100 ml, *P* = 0.837), Intensive Care Unit (ICU) stay duration (2.49 ± 2.22 days vs. 2.52 ± 2.10 days, *P* = 0.961), hospitalization duration (14 days vs. 15 days, *P* = 0.293), and chest drainage duration (12.86 ± 8.58 days vs. 13.18 ± 9.59 days, *P* = 0.886). Regarding postoperative complications, both groups had similar rates of pneumonia (*P* = 0.386), respiratory failure (*P* = 0.430), pneumothorax (*P* = 0.144), anastomotic leak (*P* = 0.356), tracheal fistula (*P* = 1.000), chylothorax (*P* = 0.268), hemorrhage (*P* = 0.993) and kidney injury (*P* = 0.989).

### Recurrence patterns

The recurrence patterns were summarized in Supplementary Table 2. The treatment failure patterns including local recurrence, distant metastasis or both. After adjustment for clinical factors, the short-interval group and long-interval group had 2.20% and 6.56% of patients experienced local recurrence, respectively (OR: 4.016, 95%CI: 0.469–34.414, *P* = 0.205), and 5.49% and 9.84% of patients experienced distant metastasis, respectively (OR: 1.686, 95CI: 0.343–8.292, *P* = 0.520). Additionally, 2.20% and 1.64% of patients experienced both local recurrence and distant metastasis simultaneously (OR: 0.403, 95%CI: 0.010–16.548, *P* = 0.632). The interval between neoadjuvant therapy and surgery showed no significant impact on the recurrence patterns.

### Survival

**Fig. 1:**
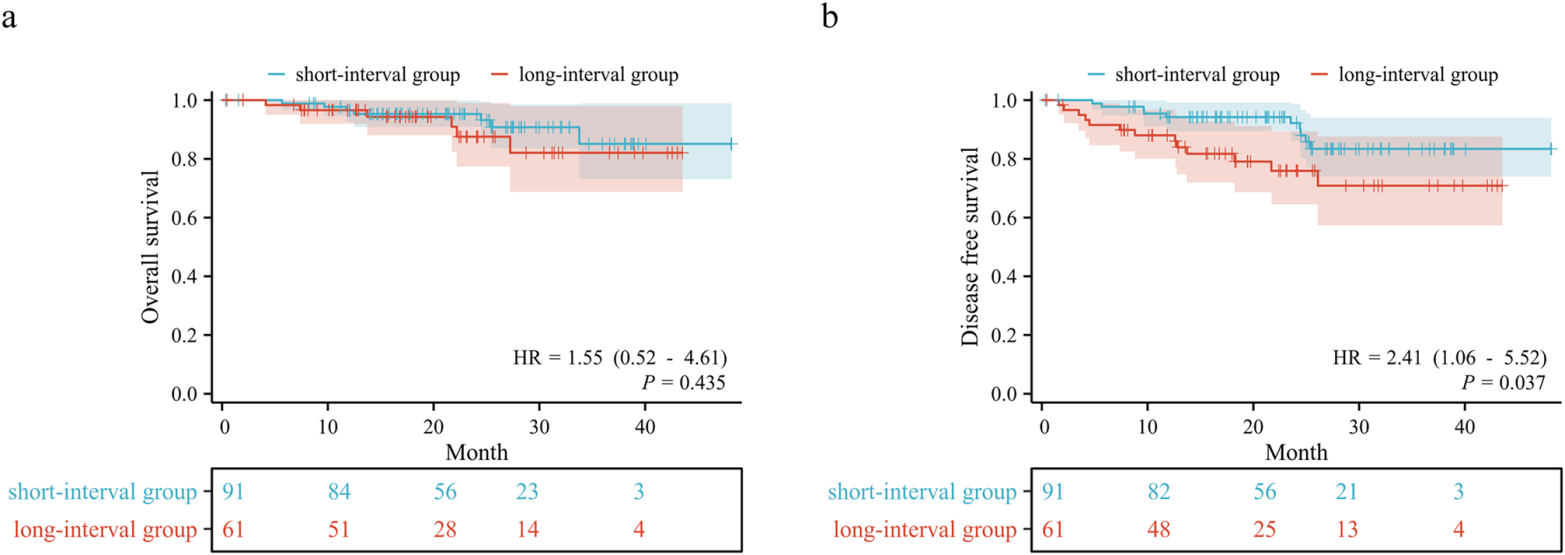
Kaplan-Meier survival analysis. **a** Comparison of OS between short-interval group and long-interval group. **b** Comparison of DFS between short-interval group and long-interval group

The survival curves for both groups were depicted in Fig. 1. The median follow-up time was 24.1 months (range: 0.3–48.2 months) in the short-interval group and 19.5 months (range: 0.5–43.5 months) in the long-interval group. The median OS was not reached in either group (HR: 1.545, 95% CI: 0.518–4.608, *P* = 0.435). The 1-year, 2-year, and 3-year OS rates were 96.52%, 95.28%, and 85.10% in the short-interval group, and 96.58%, 87.55%, and 82.07% in the long-interval group, respectively. The median DFS also did not reach in both groups (HR: 2.414, 95% CI: 1.056–5.519, *P* = 0.037). The 1-year, 2-year, and 3-year DFS rates were 94.23%, 92.18%, and 83.41% in the short-interval group, and 88.02%, 75.92%, and 70.86% in the long-interval group, respectively.

**Fig. 2:**
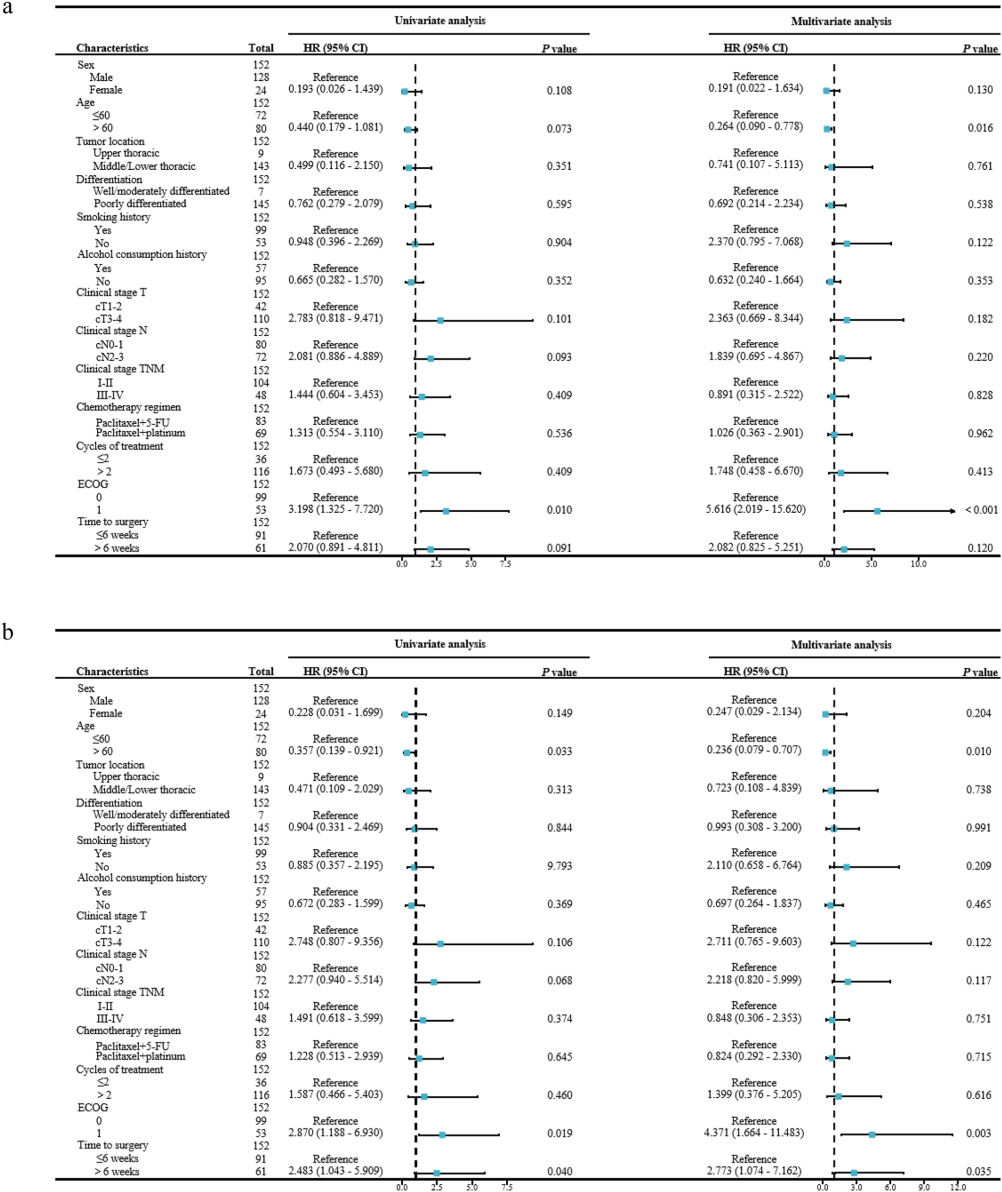
Cox proportional hazards model for univariate and multivariate analysis. **a** The association between pretreatment characteristics and OS. **b** The association between pretreatment characteristics and DFS

To further clarify whether the time to surgery is a prognostic factor, the time to surgery and clinical factors were included in both univariate and multivariate cox regression models. The findings revealed that age (HR: 0.264, 95%CI: 0.090–0.778, *P* = 0.016) and ECOG performance status (HR: 5.616, 95%CI: 2.019–15.620, *P* < 0.001) were independent predictor of OS, while the time to surgery (HR: 2.082, 95%CI: 0.825–5.251, *P* = 0.120) was not. However, in terms of DFS, age (HR: 0.236, 95%CI: 0.079–0.707, *P* = 0.010), ECOG performance status (HR: 4.371, 95%CI: 1.664–11.483, *P* = 0.003) and the time to surgery (HR: 2.773, 95%CI: 1.074–7.162, *P* = 0.035) were all independent predictor of DFS. The univariate and multivariate cox regression analyses were depicted in Fig. 2.

**Fig. 3:**
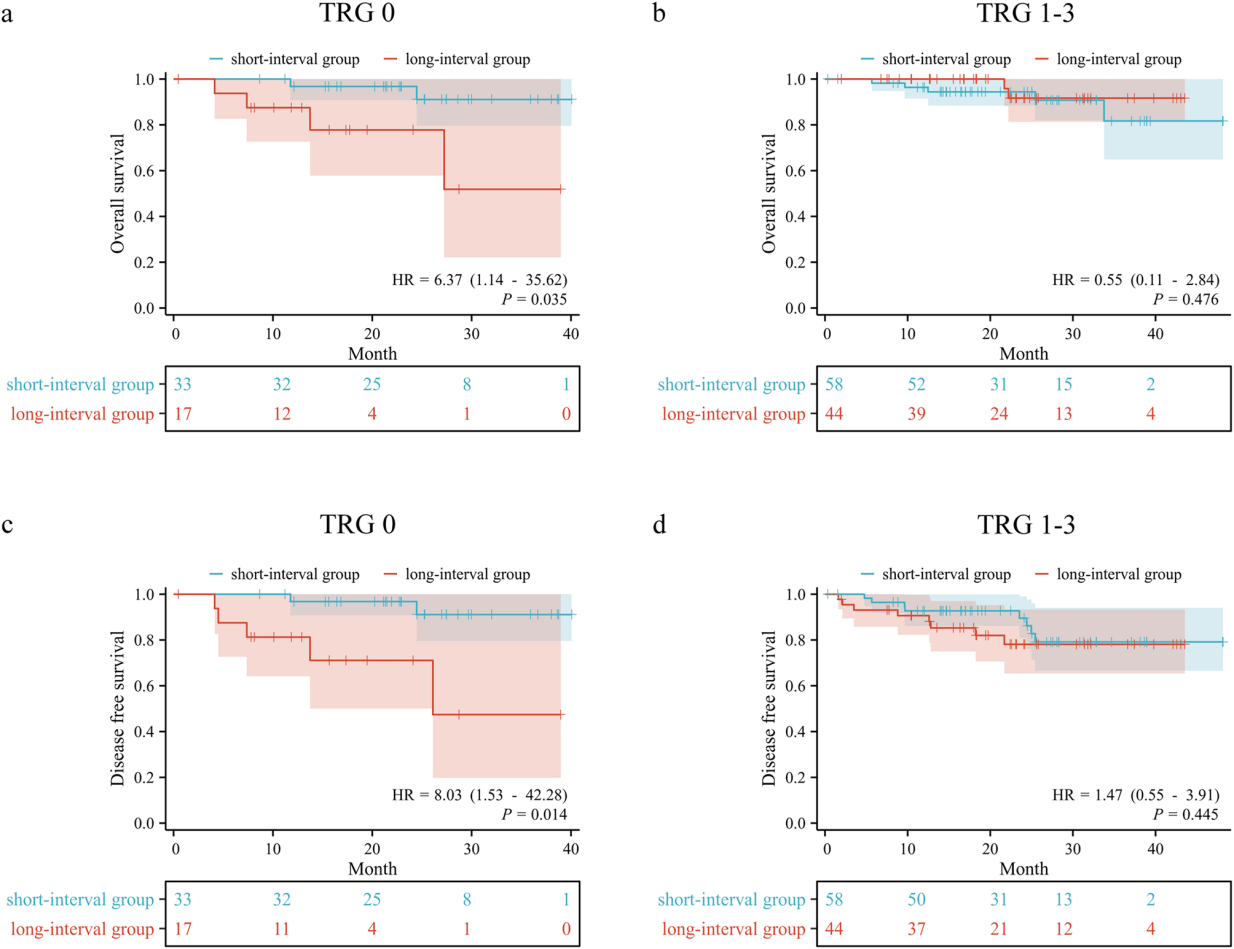
Kaplan-Meier survival analysis according to the tumor regression grade (TRG). **a** Comparison of OS between short-interval group and long-interval group for patients with TGR 0 cohort. **b** Comparison of OS between short-interval group and long-interval group for patients with TRG 1-3 cohort. **c** Comparison of DFS between short-interval group and longinterval group for patients with TGR 0 cohort. **d** Comparison of DFS between short-interval group and long-interval group for patients with TRG 1-3 cohort

According to the tumor regression grade (TRG) for subgroup analysis, in patients with a favorable response to immunotherapy (TRG 0), the short-interval group demonstrated 1-year, 2-year, and 3-year OS rates of 96.77%, 96.77%, and 91.08%, respectively, while the long-interval group showed 1-year, 2-year, and 3-year OS rates of 87.50%, 77.78%, and 51.85%, respectively. Delayed surgery significantly decreased the OS of TRG 0 cohort (HR: 6.372, 95% CI: 1.140–35.615, *P* = 0.035). In terms of DFS, the short-interval group exhibited 1-year, 2-year, and 3-year DFS rates of 96.77%, 96.77%, and 91.08%, respectively, while the long-interval group showed 1-year, 2-year, and 3-year DFS rates of 81.25%, 71.09%, and 47.40%, respectively. Delayed surgery similarly significantly reduced the DFS of TRG 0 cohort (HR: 8.031, 95%CI: 1.525–42.282, *P* = 0.014). For patients with slightly inferior pathological response (TRG 1–3), there were no statistically significant differences in OS (HR: 0.550, 95% CI: 0.107–2.840, *P* = 0.476) and DFS (HR: 1.466, 95% CI: 0.549–3.913, *P* = 0.445) between the short-interval and long-interval groups. The survival curves of different TRG were provided in Fig. 3.

## Discussion

Although the efficacy and safety of neoadjuvant PD-1 inhibitor combined with chemotherapy in locally advanced ESCC have been demonstrated in some clinical trials, the impact of the interval between immunotherapy and surgery on surgical outcomes and prognosis remains unclear. In this recent study, we compared outcomes between surgery within 6 weeks and surgery after 6 weeks following immunotherapy in the same real-world clinical practice setting. The findings revealed that delayed surgery had no significant impact on tumor downstaging, lymph node downstaging, pCR rate, and postoperative complications. However, delayed surgery was related to poorer prognosis.

Data on the optimal timing to surgery after neoadjuvant immunotherapy for ESCC are limited, but evidence on neoadjuvant chemoradiotherapy is abundant. Since the introduction of neoadjuvant chemoradiotherapy in the 1960s, it has been believed that surgery within 6 weeks is sufficient for tissue repair, resolution of tumor inflammatory response, and without causing tumor progression [18, 19]. The recommendation is to complete the surgical procedure within 4–6 weeks. Subsequent studies such as the NEOCRTEC5010 [20] and CROSS [21] trials also adopted a surgical regimen within 6 weeks after neoadjuvant treatment. Over the past few decades, a series of retrospective studies have evaluated the impact of timing to surgery after neoadjuvant chemoradiotherapy on histological response and prognosis. In studies by Lee et al. [22] and Azab et al. [23], the pCR rate increased with a prolonged interval between chemoradiotherapy and surgery, but long-term survival did not significantly improved. In a meta-analysis incorporating 10 cohort studies [24], it was found that delayed surgery did not further increase the pCR rate and OS, but rather increased the risk of anastomotic leakage. Klevebro et al. [25] similarly found no evidence to support extending the interval between chemoradiotherapy and surgery for esophageal cancer. Chiu et al. [26] reported that delayed surgery did not reduce surgical risk or increase the pCR rate, and survival was not improved either. The NeoRes II trial [27] is currently the only randomized controlled multi-center clinical trial comparing the standard time to surgery (4–6 weeks) with extended interval to surgery after neoadjuvant chemoradiotherapy for esophageal carcinoma, and the findings revealed that prolonging the surgical interval did not enhance pCR rate. Furthermore, There was a notable inclination towards poorer survival outcomes with an extended duration to surgery. These findings collectively advocate for prudence in deferring surgery following neoadjuvant chemoradiotherapy.

Compared to neoadjuvant chemoradiotherapy, immunotherapy has shown comparable efficacy in ESCC. Additionally, the tumor response patterns after immunotherapy are more diverse, with durable response patterns observed in many tumors [28]. This leads us to considered the necessity of delaying surgery in the context of immunotherapy. While clinical trials typically planed for surgery within 6 weeks after completion of immunotherapy, in real-world scenarios, surgery often occurred beyond this timeframe due to patient-related factors or the COVID-19 pandemic. In this study, we retrospectively reviewed information from patients with ESCC who underwent neoadjuvant immunotherapy combined with chemotherapy. The results revealed that delayed surgery failed to further promote primary lesion and lymph node downstaging, instead, the pCR rate was lower, although the difference was not statistically significant. This may be attributed to the relatively small sample size of the study cohort, but this trend is noteworthy, suggested that delaying surgery may entail the risk of further local lession progression.

Liang et al. found severe tissue edema and unclear tissue interspaces occurred after immunotherapy, which may increased surgical difficulty [29]. Some studies also reported that a longer waiting time after neoadjuvant treatment could lead to more severe tissue fibrosis [30]. In our short-interval and long-interval groups, there were no disparities observed between the groups concerning surgical timing or blood loss, suggested that delaying surgery did not increase surgical difficulty. Additionally, the long-interval group had numerically more lymph node dissections, which also indicated that the surgical quality of the long-interval group was at least equivalent to that of the short-interval group. Both groups also showed no differences in postoperative complications, hospitalization duration, ICU stay duration and chest drainage duration. In summary, the interval between immunotherapy and surgery did not significantly affect intraoperative procedures and postoperative recovery.

Improving prognosis is the fundamental challenge in cancer treatment. In an in vitro study involving 4T1.2 tumor-bearing mice receiving immunotherapy, prolonging the interval between immunotherapy and surgery resulted in increased mortality among surviving mice [31]. This finding underscored the importance of carefully considering the timing of surgery to achieve better oncological outcomes. Similarly, in the follow-up of our study, we found that delaying surgery did not confer any OS improved and instead led to a significant decrease in DFS. We speculated that during the delay in surgery, some lesions may continue to progress, leading to worse outcomes. Additionally, in long-interval group, the pCR rate was only 24.6%, while the pCR rate in short-interval group could reach 34.1%. pCR is a significant factor for better prognosis [32], which may also lead to poorer prognosis in long-interval group. Lastly, the main reasons for delaying surgery were poor physical condition after neoadjuvant therapy or infection with COVID-19, both of which were factors influencing prognosis [33–36].

Currently, some researchers suggested to defer surgery for patients with complete clinical response (CCR) after neoadjuvant therapy for ESCC [37, 38], similar to the watch-and-wait strategy used in rectal cancer [39], but this lacks evidence-based support. Our study results did not support this proposition. Subgroup analysis based on TRG revealed that delaying surgery in patients with excellent pathological regression (TRG 0) actually increased the risk of death and disease recurrence. This suggested that early surgery remains the optimal choice for patients who respond well to immunotherapy. Future randomized controlled trials with expanded sample sizes are warranted to further substantiate the rationale behind the watch-and-wait strategy.

To our knowledge, this study represents the most extensive investigation to date regarding the impact of the interval between immunotherapy and surgery on pathological outcomes and prognosis. Moreover, the study is grounded in real-world data, thus mitigating selection bias. Nevertheless, several limitations persist. Firstly, due to the relatively recent initiation of neoadjuvant immunotherapy research for esophageal squamous cell carcinoma, the sample size collected from a single center remains limited, which may impact the generalizability of our conclusions. In the future, it will be necessary to combine data from other medical centers to provide more comprehensive evidence. Secondly, the immunotherapeutic agents included in this study were all PD-1 antibodies, which have similar pharmacological actions and efficacy, resulting in minimal bias. However, there was significant variability in chemotherapy regimens, primarily divided into two categories: paclitaxel plus platinum and paclitaxel plus fluorouracil. This variability arises from differences in chemotherapy drug selection by different physicians when making treatment decisions. Although we adjusted for chemotherapy regimens in our analysis, there may still be some impact on the results. Thirdly, in this study, the number of neoadjuvant treatment cycles was also inconsistent. Most patients received three cycles of treatment, but a small number of patients exceeded three cycles. This inconsistency in treatment cycles may also influence the outcomes. Lastly, the follow-up period was relatively short, necessitating longer-term follow-up to validate the long-term prognosis.

## Conclusion

Delayed surgery after neoadjuvant PD-1 inhibitor combined with chemotherapy does not further improve primary tumor and lymph node downstaging or increase pCR rates; instead, it resulted in a poorer DFS. Especially for patients with a favorable response to immunotherapy, delayed surgery increases the risk of mortality and recurrence. Therefore, we recommend surgery within 6 weeks after neoadjuvant immunotherapy as prudent.

## Supplementary Information

Below is the link to the electronic supplementary material.

Supplementary file1 (DOCX 95 KB)

Supplementary file2 (DOCX 16 KB)

## Data Availability

No datasets were generated or analysed during the current study.
