# Supplementary material for: Impact of the interval between neoadjuvant immunotherapy and surgery on prognosis in esophageal squamous cell carcinoma (ESCC): a real-world study
Source: Cancer Immunol Immunother. 2024 Aug 6;73(10):202. doi: 10.1007/s00262-024-03787-2 (PMC11303633; doi:10.1007/s00262-024-03787-2)
Supplement: Supplementary file 1 — Supplementary file1 (DOCX 95 KB) [file 262_2024_3787_MOESM1_ESM.docx]

**Supplementary Table 1. The detailed dose and usage of drugs in neoadjuvant regimens.**

| Type | Drugs | Dose | Usage |
| --- | --- | --- | --- |
| PD-1 inhibitors | Camrelizumab | At a dose of 200 mg | Every 3 weeks on day 1 |
|  | Pembrolizumab | At a dose of 200 mg | Every 3 weeks on day 1 |
|  | Sintilimab | At a dose of 200 mg | Every 3 weeks on day 1 |
|  | Tislelizumab | At a dose of 200 mg | Every 3 weeks on day 1 |
|  | Toripalimab | At a dose of 240 mg | Every 3 weeks on day 1 |
|  | Penpulimab | At a dose of 200 mg | Every 3 weeks on day 1 |
|  | Nivolumab | At a dose of 360 mg | Every 3 weeks on day 1 |
| paclitaxel-based drugs | Paclitaxel | At a dose of 175 mg/m2 | Every 3 weeks on day 1 |
|  | Nab-paclitaxel | At a dose of 240 mg | Every 3 weeks on day 1 |
|  | Docetaxel | At a dose of 75 mg/m2 | Every 3 weeks on day 1 |
| platinum-based drugs | Carboplatin | At an area under the curve of 5 | Every 3 weeks on day 1 |
|  | Cisplatin | At a dose of 50 mg/m2 | Every 3 weeks on day 1 |
| fluoropyrimidine-based drugs | Capecitabine | At a dose of 1125 mg/m2 | Every 3 weeks on day 1 to 14 |
|  | S1 | At a dose of 40 mg/m2 | Every 3 weeks on day 1 to 14 |

**Supplementary Table 2. Summary of treatment failure patterns.**

| **Failure patterns** | **Short-interval group**  **(n=91)** | **Long-interval group**  **(n=61)** | **Before adjust** | | **After adjust*** | |
| --- | --- | --- | --- | --- | --- | --- |
|  |  |  | **OR (95%CI)** | ***P* value** | **OR (95%CI)** | ***P* value** |
| Regional/local | 2 (2.20%) | 4 (6.56%) | 3.123 (0.703-16.710) | 0.219 | 4.016 (0.469-34.414) | 0.205 |
| Distant | 5 (5.49%) | 6 (9.84%) | 1.876 (0.514-5.730) | 0.350 | 1.686 (0.343-8.292) | 0.520 |
| Both | 2 (2.20%) | 1 (1.64%) | 0.742 (0.050-6.500) | 1.000 | 0.403 (0.010-16.548) | 0.632 |

*The multivariate logistic regression model adjusted age, sex, tumor location, differentiation, smoking history, alcohol consumption history, ECOG performance status, clinical T stage, clinical N stage, clinical TNM stage, cycles of neoadjuvant treatment and the regimen of chemotherapy.


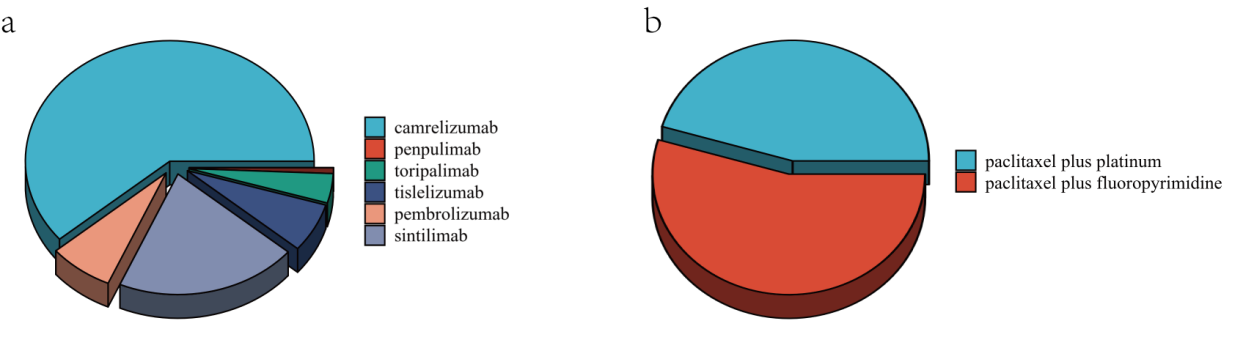


**Supplementary Figure 1. The regimen of neoadjuvant treatment. a) The regimen of PD-1 inhibitors in the total cohort. b) The regimen of chemotherapy in the total cohort.**
