# Supplementary material for: Impact of the interval between neoadjuvant immunotherapy and surgery on prognosis in esophageal squamous cell carcinoma (ESCC): a real-world study
Source: Cancer Immunol Immunother. 2024 Aug 6;73(10):202. doi: 10.1007/s00262-024-03787-2 (PMC11303633; doi:10.1007/s00262-024-03787-2)
Supplement: Supplementary file 2 — Supplementary file2 (DOCX 16 KB) [file 262_2024_3787_MOESM2_ESM.docx]

**Supplementary Table 1. The surgery outcomes between short-interval group and long-interval group.**

| **Surgery outcomes** | **Short-interval group**  **(n=91)** | **Long-interval group**  **(n=61)** | ***P* value** | **Adjusted *P* value*** |
| --- | --- | --- | --- | --- |
| R0 resection rate | 91 (100.0%) | 61 (100.0%) | 1.000 | 1.000 |
| Lymph node resection number | 40.34±16.77 | 44.51±21.13 | 0.306 | 0.133 |
| Positive lymph node number | 0.70±1.82 | 0.74±1.28 | 0.596 | 0.887 |
| Operation time/min | 325.13±141.48 | 340.30±191.10 | 0.540 | 0.689 |
| Blood loss/ml | 100 (IQR: 50-300) | 100 (IQR: 50-400) | 0.028 | 0.837 |
| Hospitalization duration/day | 14 (IQR: 10-77) | 15 (IQR: 9-100) | 0.616 | 0.293 |
| ICU stay duration/day | 2.49±2.22 | 2.52±2.10 | 0.932 | 0.961 |
| Chest drainage duration/day | 12.86±8.58 | 13.18±9.59 | 0.294 | 0.886 |
| Pneumonia | 3 (3.30%) | 5 (8.20%) | 0.268 | 0.386 |
| Respiratory failure | 7 (7.69%) | 3 (4.92%) | 0.741 | 0.430 |
| Pneumothorax | 1 (1.10%) | 3 (4.92%) | 0.303 | 0.144 |
| Anastomotic leakage | 19 (20.88%) | 9 (14.75%) | 0.398 | 0.356 |
| Tracheal fistula | 1 (1.10%) | 0 (0.00%) | 1.000 | 1.000 |
| Chylothorax | 2 (2.20%) | 2 (3.28%) | 1.000 | 0.268 |
| Hemorrhage | 1 (1.10%) | 0 (0.00%) | 1.000 | 0.993 |
| Kidney injury | 1 (1.10%) | 1 (1.64%) | 1.000 | 0.989 |
| Perioperative death | 0 (0.00%) | 0 (0.00%) | 1.000 | 1.000 |

*The multiple linear regression models or multivariate logistic regression model adjusted age, sex, tumor location, differentiation, smoking history, alcohol consumption history, ECOG performance status, clinical T stage, clinical N stage, clinical TNM stage, cycles of neoadjuvant treatment and the regimen of chemotherapy.
